# Whole-Blood PCR Preferred for Timely Diagnosis of Neuroinvasive West Nile Virus Infections: Lessons From the 2021 Arizona Outbreak

**DOI:** 10.1093/ofid/ofae188

**Published:** 2024-04-24

**Authors:** Sabirah Kasule, Emily Fernholz, Leah Grant, Amy Kole, Thomas E Grys, Erin Kaleta, Elitza S Theel, Bobbi Pritt, Erin H Graf

**Affiliations:** Division of Infectious Diseases, Department of Internal Medicine, Mayo Clinic, Phoenix, Arizona, USA; Division of Infectious Disease, Department of Internal Medicine, BronxCare Health System, Bronx, New York, USA; Department of Laboratory Medicine and Pathology, Mayo Clinic, Rochester, Minnesota, USA; Division of Infectious Diseases, Department of Internal Medicine, Mayo Clinic, Phoenix, Arizona, USA; Division of Infectious Diseases, Department of Internal Medicine, Mayo Clinic, Phoenix, Arizona, USA; Department of Laboratory Medicine and Pathology, Mayo Clinic, Phoenix, Arizona, USA; Department of Laboratory Medicine and Pathology, Mayo Clinic, Phoenix, Arizona, USA; Department of Laboratory Medicine and Pathology, Mayo Clinic, Rochester, Minnesota, USA; Department of Laboratory Medicine and Pathology, Mayo Clinic, Rochester, Minnesota, USA; Department of Laboratory Medicine and Pathology, Mayo Clinic, Phoenix, Arizona, USA

**Keywords:** arboviral infections, laboratory-developed tests, molecular diagnostics, neuroinvasive West Nile virus, West Nile virus

## Abstract

**Background:**

In 2021, the state of Arizona experienced the largest focal outbreak of West Nile virus (WNV) in US history. Timely and accurate diagnostic testing remains a challenge for WNV due to transient viremia and limited immunoassay specificity. Recent studies have identified whole blood (WB) and urine as more sensitive specimen types for the detection of WNV RNA.

**Methods:**

We evaluated ordering practices, test performance, and patient characteristics of probable and confirmed cases. In total, we identified 190 probable and proven cases, including 127 patients (66.8%) with neuroinvasive disease.

**Results:**

Among all cases, only 29.5% had WNV polymerase chain reaction (PCR) testing ordered on WB, of which 80.3% resulted as positive, including 7 cases in which WNV serologic testing was negative and 5 cases for which serologic testing was not ordered. In comparison, only 23.7% of cases that had cerebrospinal fluid (CSF) PCR ordered had a positive result, including 3 cases that were negative by PCR on WB. In contrast, WNV PCR on WB detected 12 neuroinvasive cases that were CSF PCR negative. WNV PCR testing in urine was only ordered on 2 patients, both of whom were positive. Crossing cycle threshold (Ct) values were not significantly different between WB and CSF specimen types, nor was there a correlation between Ct value and days from symptom onset at the time of sample collection; all specimen types and time points had Ct values, with 98% above 30. WB was positive by WNV PCR in several patients for >7 days (range, 7–25 days) after symptom onset, as was the CSF PCR.

**Conclusions:**

Taken together, these findings indicate that WNV PCR testing on WB may be the best initial test for timely diagnosis of WNV infection, irrespective of clinical manifestation; however, if negative in patients with suspected neuroinvasive disease, WNV PCR testing on CSF should be ordered.

West Nile virus (WNV) was first isolated in culture in the 1930s from the blood of a febrile woman in Uganda and was subsequently found to cause sporadic human outbreaks in Africa, Asia, and Europe [1]. It was not until 1999 that a cluster of human cases was discovered in New York City, documenting expansion of the virus to the Western Hemisphere [2, 3]. WNV is an arboviral member of the family *Flaviviridae* and is most commonly transmitted by *Culex* mosquitos [4]. The reservoirs for WNV are thought to be various wild birds that may symptomatically or asymptomatically transmit the virus between each other, primarily through a mosquito intermediate vector [5]. Although the initial cases of WNV in the United States occurred in the Northeast, over the last 2 decades the majority of cases have been documented in the Southwestern United States, Nebraska, and the Dakotas. In 2021, Arizona had the largest identified focal WNV outbreak in US history [6, 7]. The reasons for this new peak in human transmission have yet to be fully elucidated but likely relate to an above-average rainy season and subsequent mosquito population blooms, as well as alterations in bird migration patterns [8].

WNV is a nationally reportable disease, but incidence is likely underreported due to lack of health care provider awareness, limited availability and limitations of diagnostic testing, and high rates of asymptomatic infection. Approximately 20% of transmissions result in symptomatic infection including headache, fever, gastrointestinal discomfort, and/or a maculopapular, erythematous rash [9–11]. Less than 1% of infections result in neuroinvasive disease [12]. Central nervous system infection manifests on a spectrum of presentations from headache to myelitis, encephalopathy, multi-organ failure, and death [13]. Individuals who survive neuroinvasive disease often have long-lasting neurologic and functional deficits [14]. Older age (>65 years) and immunosuppression are established risk factors for neuroinvasive disease, but cases can occur in any age or population [15]. There are currently no approved medications or specific guidelines for the treatment of neuroinvasive WNV infection.

Diagnosis of WNV infection is challenging. Viral particles circulate in the bloodstream and CSF for a limited time, leading to poor molecular test sensitivity [16–18]. Serologic testing, the mainstay of WNV diagnosis and surveillance, offers high sensitivity after the first 5 days of symptoms, although immunosuppressed patients may remain seronegative for longer [19]. Additionally, serologic cross-reactivity with antibodies to other closely related flaviviruses (eg, St. Louis encephalitis virus, which is present in many WNV-endemic regions) may occur, requiring additional confirmatory testing to be performed (eg, plaque reduction neutralization test), which can lead to delays in definitive diagnoses [20].

Whole blood (WB) and urine have recently been described, in small studies, as more sensitive specimen types for the detection of WNV RNA [16, 21]. Fractionated red blood cells have been shown to carry higher WNV viral loads compared with matched plasma, and WNV is hypothesized to adhere to red blood cells [21]. As both urine and whole-blood sources were available for routine patient testing via our reference laboratory in 2021, we evaluated ordering patterns, results, and patient characteristics for all confirmed and probable WNV cases seen at Mayo Clinic Arizona during the 2021 outbreak.

## METHODS

### Patient Selection

All Mayo Clinic Arizona patients who had a positive test result for WNV antibodies and/or WNV nucleic acid between June and December 2021 were included. Electronic health records (EHRs) were reviewed to determine patient characteristics including age, sex, race, immunosuppression status (including, but not limited to having HIV, underlying malignancy, history of a solid organ or hematopoietic stem cell transplant, and active receipt of chemotherapy or other immunomodulatory therapy), and clinical diagnosis of WNV infection with or without neuroinvasive disease. Outcome data that were evaluated included (1) duration of symptoms before positive test results, (2) requirement for hospital and/or intensive care unit (ICU) admission, (3) duration of symptoms before hospitalization, and (4) outcomes related to lack of neurologic improvement or death. Cases were considered proven or probable for WNV infection based on the Centers for Disease Control and Prevention (CDC) case definitions (reference PMID 33661868). Briefly, a positive WNV PCR was considered proven infection, and a positive anti-WNV IgM result in serum and/or CSF was considered probable infection, as confirmatory IgM testing was sent to a public health laboratory and results were not scanned into the EHR. Cases in which the only diagnostic testing that was positive was anti-WNV immunoglobulin G (IgG) in serum or CSF were not considered diagnostic and were excluded. Patients were considered to have proven neuroinvasive disease if the WNV PCR was positive from any source if they presented with compatible neurologic symptoms and had a clinical diagnosis of neuroinvasive WNV indicated in the EHR. A patient was considered to have probable neuroinvasive disease if WNV IgM was positive from any source with compatible neurologic symptoms and a clinical diagnosis of neuroinvasive WNV was indicated in the EHR.

### Diagnostic Testing

#### WNV Serologic Testing

Serologic testing for anti-WNV IgM and IgG was performed as part of routine patient care via Mayo Clinic Laboratories (MCL) in Rochester, Minnesota, using the WNV IgG DxSelect and WNV IgM Capture DxSelect (DiaSorin Molecular, Cypress, CA, USA) enzyme-linked immunosorbent assays (ELISAs) per manufacturer instructions. This includes background subtraction testing for all initially WNV IgM–reactive samples to rule out the possibility of false-positive results. All testing was performed on the Agility ELISA processor (Dynex, Chantilly, VA, USA). These assays have been cleared by the Food and Drug Administration (FDA) for use in serum; testing of CSF using these assays was validated as an off-label source in accordance with the Clinical Laboratory Improvement Amendments as a laboratory-developed test (LDT).

#### Molecular Testing

Molecular testing was also performed, when ordered, as part of routine patient care via MCL. Nucleic acid extraction was performed on the NucliSENS easyMAG (bioMérieux, Marcy-l'Étoile, France). RT-PCR was performed on the LightCycler 480 II (Roche, Basel, Switzerland) using the RealStar WNV RT-PCR Kit 2.0 (Altona Diagnostics, Hamburg, Germany) following the manufacturer's instructions for use for master mix volumes and thermocycling conditions. RT-PCR testing has been validated by MCL as an LDT for the detection of WNV RNA in EDTA-preserved whole blood, CSF, and urine, among other sample types. Cycle threshold (Ct) values were recorded for all PCR-positive patients. The limit of detection, in genomic targets per microliter, was established to be 44.3 in whole blood (average Ct, 37.8), 5.23 in urine (average Ct, 35.28), and 1.28 in CSF (average Ct, 37.01). For 19 days during the study, specimens were sent to Quest diagnostics for their proprietary laboratory-developed PCR testing due to reagent shortages in the primary laboratory. As whole blood was not an acceptable source, only plasma and/or CSF was sent to Quest; therefore, these data were excluded from the analysis as Ct values were not available.

### Statistical Analysis

Median values, interquartile ranges, Mann-Whitney tests, 2-way analysis of variance (ANOVA), and Spearman correlations were all calculated using GraphPad Prism, version 8.

## RESULTS

Over the study time period, 190 cases of probable or proven WNV infection were identified. Basic demographic information is included in Table 1. Most patients were male (63%) and White (95%), with a median age (range) of 65 (6–94) years. Twenty-three percent (43/190) of patients were immunocompromised, including solid organ transplant recipients (n = 15), individuals receiving chemotherapy for malignancies (n = 14), individuals receiving immunomodulatory agents for autoimmune conditions (n = 9), splenectomized individuals (n = 2), individuals with HIV (n = 2), and a stem cell transplant recipient (n = 1). Sixty-seven percent (127/190) of WNV cases were considered neuroinvasive, with 75.2% of all patients requiring hospitalization and 13.9% of those requiring ICU admission. Overall, 7 (5.5%) cases experienced persistent neurologic complications (at the time of this writing), and a separate 7 patients (5.5%) died. Of the WNV infections in immunocompromised hosts, 22.4% were neuroinvasive, similar to overall neuroinvasive cases. However, immunocompromised hosts represented a higher proportion of cases with severe complications including myelitis (45.8%), ICU admission (45%), and death (85.7%).

**Table 1.** Summary of Characteristics of Patients Diagnosed With West Nile Virus Infection, n = 190

Characteristic	No. (% or Range)
Sex, male	120 (63)
Median age, y	65 (6–94)
Race, Caucasian	180 (95)
Immunocompromised	43 (22.6)
Transplant recipient	15 (8)
Duration of symptoms before presentation (admitted and nonadmitted), d	5 (0–33)
Required hospital admission	143 (75.2)
Duration of symptoms before admission (admitted), d	5 (0–33)
Duration of hospitalization, d	6 (0–64)
Required ICU admission (% of those admitted)	20 (13.9)
Confirmed WNV case^a^	51 (26.8)
Probable WNV case^a^	139 (73.2)
Neuroinvasive WNV disease	127 (66.8)
Severe neurologic sequalae (of neuroinvasive cases above)	7 (5.5)
Died (of neuroinvasive cases above)	7 (5.5)
WNV PCR testing ordered (any source)	72 (37.9)
WNV serologic testing ordered (any source)	176 (92.6)
WNV PCR and serologic testing ordered (any source)	71 (37.4)

^a^Based on CDC criteria.

Abbreviations: CDC, Centers for Disease Control and Prevention; ICU, intensive care unit; WNV, West Nile virus.

Diagnostic test ordering was not standardized at the time of the study; thus there was significant heterogeneity in what testing was ordered for each patient. The majority of cases were identified via serologic testing (92.6%), resulting in 73.2% of cases defined as probable per CDC criteria (Table 1). A smaller subset of cases had WNV PCR testing performed from any source (37.9%), resulting in 26.8% of cases meeting CDC criteria for proven WNV infection. WNV PCR testing from WB yielded the highest number of confirmed cases (88.2%), while PCR testing of CSF, when ordered, contributed to 9.8% of confirmed cases. Testing of urine was only sent in 2 cases, contributing to 3.9% of confirmed cases.

Although the majority of patients with WNV did not have WB submitted for PCR testing (n = 134, 70.5%), PCR testing of this sample type was associated with the largest number of confirmed WNV cases (88.2%). As a result, for the purposes of this study, data were analyzed based on whether WNV PCR testing was ordered and what the reported results were (Table 2). Among WB WNV PCR–positive cases, 15.6% of patients were seronegative, while 11.1% did not have serologic testing ordered, indicating that WNV PCR on WB was the primary diagnostic test in over a quarter of confirmed cases (Table 2). Among seronegative patients, 71.4% were immunocompromised, and 42.8% had serial serum antibody testing performed, with all showing IgM seroconversion 5 to 14 days after the initial positive PCR result. WNV PCR testing on CSF was concurrently positive in 8.9% of WB PCR–positive cases and negative in 26.7% of WB PCR–positive cases. Importantly, all WB PCR–positive, CSF PCR–negative cases were clinically diagnosed with neuroinvasive disease, suggesting that PCR testing on WB is more sensitive for diagnosis of neuroinvasive WNV infection as compared with PCR testing of CSF. Conversely, of the 11 patients with clinically diagnosed WNV infections who had a negative WB PCR test, 3 (27%) had a positive CSF PCR and a diagnosis of WNV neuroinvasive disease. Two of these patients were seronegative, both were immunocompromised, and 1 patient received follow-up testing that demonstrated IgM seroconversion 14 days after symptom onset. Of the 134 cases that did not have WB WNV PCR ordered, 16 (11.9%) had CSF PCR testing ordered, with 2 positive results (Table 2).

**Table 2.** WNV Laboratory Test Results Stratified by WB PCR

Test Result^a^	Positive WB PCR, n = 45	Negative WB PCR, n = 11	WB PCR Not Ordered, n = 134
	No. (%)	No. (%)	No. (%)
Neuroinvasive cases (diagnosed clinically)	30 (66.7)	10 (90.9)	82 (61.2)
Positive serum IgM & IgG	16^b^ (35.6)	3 (27.3)	65^b^ (48.5)
Positive serum IgM only	17^c^ (37.8)	5^c^ (45.5)	53 (39.6)
Positive serum IgG only^d^	0 (0)	0 (0)	1^d^ (0.8)
Negative serum IgM & IgG	7^e^ (15.6)	2^f^ (18.2)	4^f^ (3.0)
Serologic testing not ordered in serum	5 (11.1)	1^f^ (9.1)	8^f^ (6.0)
Positive CSF IgM & IgG	9^b^ (20)	3^b^ (27.3)	29^b^ (21.6)
Positive CSF IgM only	18 (40)	3 (27.3)	34^c^ (25.4)
Negative CSF IgM & IgG	4 (8.9)	3 (27.3)	4 (3.0)
Serologic testing not ordered in CSF	14 (31.1)	2 (18.2)	67 (50.0)
Positive CSF PCR	4 (8.9)	3 (27.3)	2 (1.5)
Negative CSF PCR	12 (26.7)	3 (27.3)	14 (10.4)
CSF PCR not ordered	29 (64.4)	5 (45.5)	118 (88.1)
Positive urine PCR	1 (2.2)	0 (0)	1 (0.8)
Urine PCR not ordered	44 (97.8)	11 (100)	133 (99.2)

Abbreviations: CSF, cerebrospinal fluid; IgG, immunoglobulin G; IgM, immunoglobulin M; PCR, polymerase chain reaction; WB, whole blood; WNV, West Nile virus.

^a^Collected within 48 hours of whole-blood PCR test.

^b^Includes equivocal IgG.

^c^Includes equivocal IgM.

^d^Only included as a case if other testing was supportive (positive CSF IgM, n = 1).

^e^For these 7 seronegative specimens, the time at which antibody testing was ordered ranged from 2 days to 16 days, with a median of 4 days.

^f^Diagnosed via CSF testing (positive PCR, n = 4; positive CSF IgM, n = 10), or urine PCR (n = 1).

WNV serologic testing in CSF was ordered in 65% of cases (Table 2). Among all WNV cases, 5.8% were diagnosed solely via detection of anti-WNV IgM in CSF. This included 9 patients for whom serum-based serologic testing and WB PCR testing were not ordered and 2 serum-seronegative patients who were not immunocompromised and for whom WB PCR testing was not ordered.

**Figure 1.**
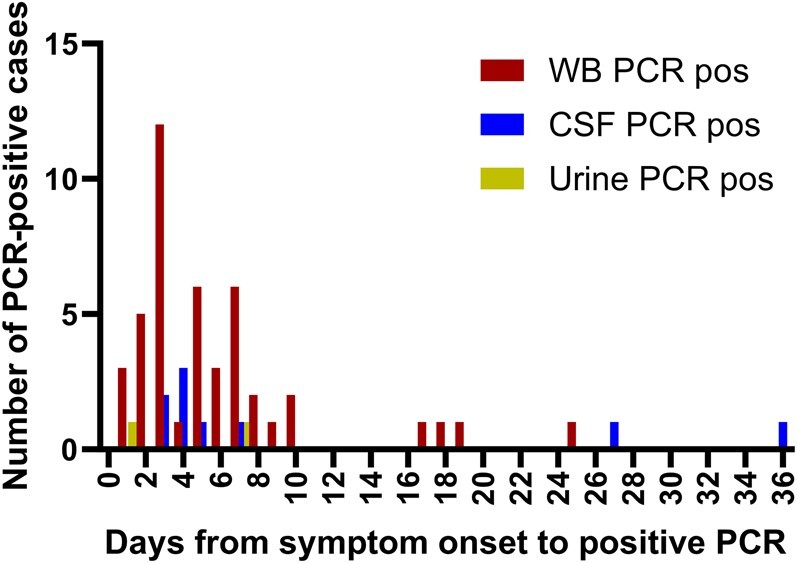
WNV PCR–positive cases by days from symptom onset. Histogram showing WNV PCR–positive cases detected in WB (red), CSF (blue), and urine (yellow) by days after symptom onset. Abbreviations: CSF, cerebrospinal fluid; PCR, polymerase chain reaction; WB, whole blood; WNV, West Nile virus.

**Figure 2.**
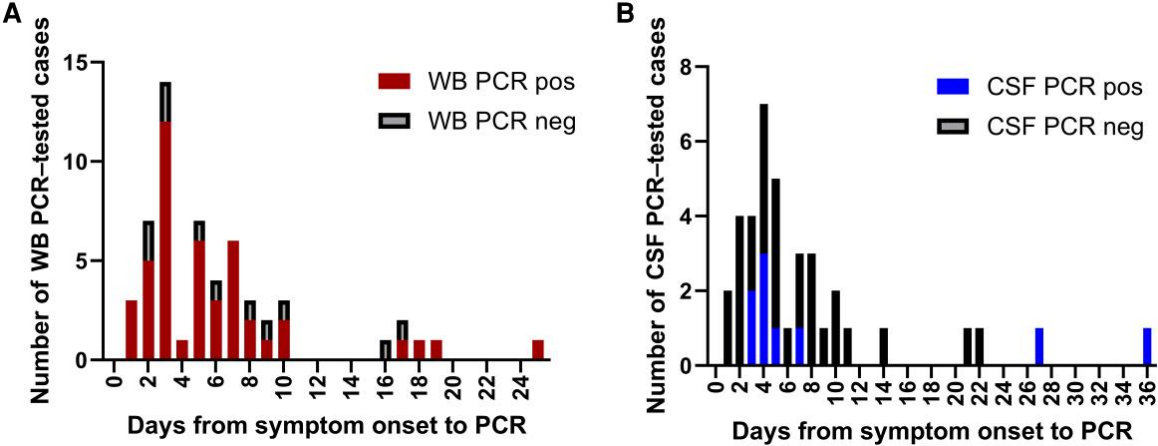
WNV cases tested by PCR by days from symptom onset. Stacked histograms showing the number of cases that were tested by PCR in whole blood, WB (A), and/or CSF (B). Positive PCR results are shown in color; negative PCR results are shown in black. Abbreviations: CSF, cerebrospinal fluid; PCR, polymerase chain reaction; WB, whole blood; WNV, West Nile virus.

To further investigate the diagnostic yield of WNV PCR testing in WB, we compared PCR positivity relative to the duration of symptoms before sample collection (Figure 1). Eighty percent of WB PCR–positive cases (36/45) tested positive ≤7 days before the onset of symptoms (median, 5 days); however, several patients tested positive over a week after symptom onset (n = 9). CSF PCR–positive patients had a similar median time from symptom onset to test positivity (median, 4 days) (Figure 1), with CSF from 2 patients (22.2% of positive cases) testing PCR positive >7 days after symptom onset. Only 2 patients were PCR-positive from urine, and both tested positive within 7 days of symptom onset. Figure 2 displays the overall PCR test results from all patients diagnosed with WNV who had a WB PCR ordered and/or a CSF PCR ordered. The number of days from symptom onset to test ordering was not significantly different between the 2 sources; however, the percent PCR positivity in WB was significantly higher compared with CSF (*P* < .05, 2-way ANOVA).

**Figure 3.**
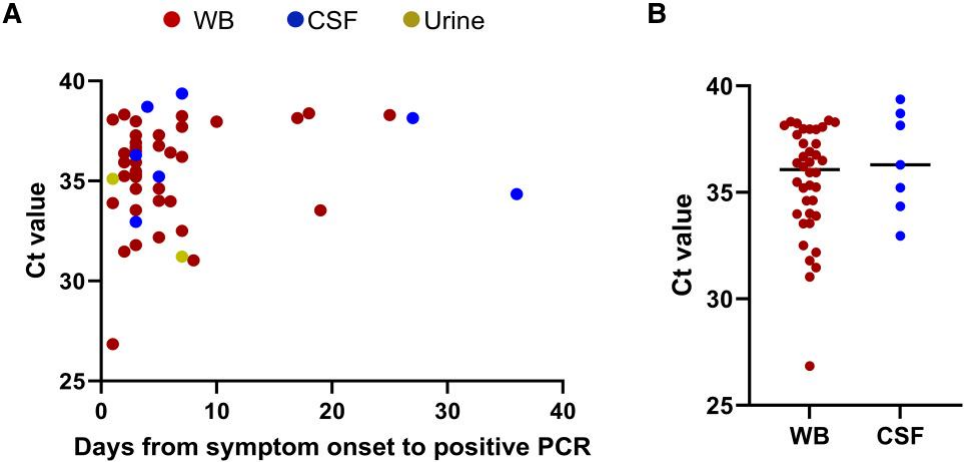
WNV PCR Ct values compared with days from symptom onset and between specimen types. A, Crossing Ct values from whole blood (red dots, n = 38), CSF (blue dots, n = 7), and urine (yellow dots, n = 2) PCR–positive specimens are displayed on the y-axis compared with days from symptom onset to positive PCR on the x-axis. Spearman correlation was not significant for all data points analyzed together or separately for WB and CSF. B, Ct values displayed on the y-axis vs specimen type on the x-axis. Median values are displayed by the black line, and interquartile ranges via the colored lines. Mann-Whitney *P* value was not significant. Abbreviations: CSF, cerebrospinal fluid; Ct, cycle threshold; PCR, polymerase chain reaction; WB, whole blood; WNV, West Nile virus.

To determine whether any correlation exists between days from symptom onset and relative quantity of WNV RNA, we used PCR Ct values from WNV-positive patients as an approximate surrogate of viral RNA quantity since the assay input and output volumes are all standardized. There was no correlation between PCR Ct values and days post–symptom onset irrespective of whether WB- and CSF-positive patients were evaluated together or separately (Spearman correlation *P* values not significant) (Figure 3). The median Ct value for WB PCR–positive specimens was not significantly different from CSF (Mann-Whitney *P* value not significant) (Figure 3), and with only 2 positive urine specimens, Ct values were not compared for this sample type. Importantly, the median Ct values (IQR) were very high at 36.07 (33.96–37.77) and 36.29 (34.34–38.71) for WB and CSF, respectively, suggesting that samples may be bordering on the limit of detection for the majority of confirmed WNV cases.

## DISCUSSION

Timely and accurate diagnosis of WNV infection is important for patient management, public health surveillance, and continued research on potential therapeutics to combat neuroinvasive disease and death. Yet the optimal approaches to diagnostic testing for WNV have yet to be well defined. In the present study, we identified 190 patients with proven or probable WNV disease during a single large outbreak in Arizona [7]. While only WNV serologic testing was ordered in the majority of these cases, patients for whom WNV PCR testing was ordered from WB showed a high positivity rate (80.3% of all WNV cases were sent for whole-blood PCR testing). In addition, several patients had negative serum WNV antibody results, the majority of whom were immunocompromised. Combined, these data suggest that WNV PCR performed on WB may be a more sensitive approach for timely diagnosis of WNV infection, particularly in immunocompromised patients, as compared with serologic testing alone. Our findings are consistent with a recent study that demonstrated detection of WNV RNA in 33/35 (87%) WB samples compared with 20/77 (26%) serum samples and 11/66 (16.6%) CSF samples in a cohort of proven and probable WNV cases [17]. This study also detected WNV RNA in 28/48 (58%) urine samples, consistent with another recent study [16]. WNV PCR testing on urine was ordered and positive in only 2 patients in our study, and thus we could not adequately evaluate the value of this sample type. WNV RNA was detected in CSF from patients clinically diagnosed with neuroinvasive disease in 23.7% of those tested, notably higher than prior studies that estimated sensitivity at 10%–15% [16–18]. In our study, 3 cases of confirmed neuroinvasive disease had WNV RNA detectable only in CSF and not in WB. Thus, when CSF is available, WNV PCR testing of CSF in a patient with compatible neuroinvasive disease symptoms should be pursued, even if WB is negative. However, CSF PCR should not be ordered without whole-blood PCR due to the lack of sensitivity demonstrated by this and other studies, even at early time points after symptom onset. More than half of the cases in this study had WNV antibody testing performed on CSF, which led to the detection of 2 patients with neuroinvasive disease who were seronegative in blood, with WNV RNA not detected in either WB or CSF. As a result, testing for WNV antibodies in CSF from patients with compatible symptoms and otherwise negative test results remains of value, particularly during WNV outbreaks.

Our study also showed that WNV PCR Ct values were high in almost all PCR-positive cases. The median Ct values for WB and CSF were >35 cycles, with only 1 patient having a Ct value <30. There was no significant difference in Ct values between WB and CSF, and there was no correlation between Ct values and duration of symptoms at the time of testing. WNV RNA was also detected in several patients tested over a week after symptom onset. Most of these PCR-positives were in WB specimens, which challenges the conventional dogma, in the referenced citations, that viremia is undetectable 5–7 days after symptom onset [22, 23]. Lustig and colleagues recently hypothesized that while WNV PCR sensitivity is maximal in the early acute window, WB PCR testing may maintain up to 50% sensitivity at 3 weeks post–symptom onset [18]. Taken together, our data support this hypothesis, but also highlight that positive results approached the limit of detection of the assay employed at all time points after symptom onset.

Our study has several important limitations, including that these data were retrospectively acquired from a single center during a single WNV season from a cohort of patients who were 95% Caucasian, which may limit broad applicability. Second, our overall proportion of neuroinvasive WNV cases was more than half of the total study population. Thus, the sensitivity of PCR on WB may be skewed by the more severe disease presentation, although one could argue that this is the population that most urgently needs accurate and timely testing. Third, test ordering was not standardized during the study period, and WNV PCR was ordered on less than half of all cases. Additional studies are needed to fully assess the sensitivity of WNV PCR testing on WB and urine as compared with other specimen types and serologic testing, as well as specificity, as all positive results had high Ct values, which could raise concern for false positivity. Finally, it is important to note that there are no FDA-approved assays for the molecular detection of WNV outside of blood product screening. Several reference laboratories offer WNV PCR via LDTs; however, as of the time of this writing, most appear to have validated assay performance for only the conventional specimen types (ie, plasma and CSF), which might limit the widespread applicability of our findings. Select local and state public health laboratories may also offer molecular testing for WNV using LDTs, but may limit testing to plasma or serum. Thus, health care providers and laboratorians should consider the best send-out approach and specimen types for their clinical practice.

Climate change is predicted to impact the geographic and seasonal patterns of arboviral infections, including for WNV [24]. Given the lack of effective therapies combined with a large number of WNV cases in 2021, expanded awareness and accurate diagnosis are critically important to support continued infection control efforts and research on treatment. We suggest that WB and possibly urine are the preferable specimen types for detection of WNV by PCR, followed by testing of CSF in patients with compatible symptoms but who are PCR-negative in other sample types. Serologic testing for WNV will continue to play a diagnostic role in the diagnosis of this arboviral infection, particularly in patients who are PCR-negative; however, the sensitivity of this method remains low during the acute phase of disease.
